# Development of a Novel 3D Tumor-tissue Invasion Model for High-throughput, High-content Phenotypic Drug Screening

**DOI:** 10.1038/s41598-018-31138-6

**Published:** 2018-08-29

**Authors:** T. J. Puls, Xiaohong Tan, Mahera Husain, Catherine F. Whittington, Melissa L. Fishel, Sherry L. Voytik-Harbin

**Affiliations:** 1 Weldon School of Biomedical Engineering, Purdue University, West Lafayette, IN 47907 USA; 2 Department of Oncology, Eli Lilly and Company, Indianapolis, IN 46285 USA; 3 Department of Pediatrics, Wells Center for Pediatric Research, Indiana University School of Medicine, Indianapolis, IN 46202 USA; 4 Department of Pharmacology and Toxicology, Indiana University School of Medicine, Indianapolis, IN 46202 USA; 5 Pancreatic Cancer Signature Center, Indiana University Simon Cancer Center, Indianapolis, IN 46202 USA; 6 Department of Basic Medical Sciences, Purdue University, West Lafayette, IN 47907 USA

## Abstract

While much progress has been made in the war on cancer, highly invasive cancers such as pancreatic cancer remain difficult to treat and anti-cancer clinical trial success rates remain low. One shortcoming of the drug development process that underlies these problems is the lack of predictive, pathophysiologically relevant preclinical models of invasive tumor phenotypes. While present-day 3D spheroid invasion models more accurately recreate tumor invasion than traditional 2D models, their shortcomings include poor reproducibility and inability to interface with automated, high-throughput systems. To address this gap, a novel 3D tumor-tissue invasion model which supports rapid, reproducible setup and user-definition of tumor and surrounding tissue compartments was developed. High-cell density tumor compartments were created using a custom-designed fabrication system and standardized oligomeric type I collagen to define and modulate ECM physical properties. Pancreatic cancer cell lines used within this model showed expected differential invasive phenotypes. Low-passage, patient-derived pancreatic cancer cells and cancer-associated fibroblasts were used to increase model pathophysiologic relevance, yielding fibroblast-mediated tumor invasion and matrix alignment. Additionally, a proof-of-concept multiplex drug screening assay was applied to highlight this model’s ability to interface with automated imaging systems and showcase its potential as a predictive tool for high-throughput, high-content drug screening.

## Introduction

Despite progress in treating some cancers, metastatic tumors remain nearly impossible to treat, thus metastasis continues to be the predominant cause of cancer-related deaths^1^. This problem is especially apparent for highly metastatic cancers like pancreatic ductal adenocarcinoma (PDAC), where approximately 90% of patients present with invasive or metastatic disease^2^. While limited treatment options for patients with metastases represents a multi-facetted problem, one major shortcoming is the lack of predictive preclinical models of invasive tumor phenotypes that can be used for mechanistic studies, biomarker and drug target identification, and drug screening^1,3^. Since the initial step in the tumor metastatic process involves tumor cell engagement, remodeling, and invasion of the surrounding tissue extracellular matrix (ECM), it is becoming increasingly clear that accurate recreation of such three-dimensional (3D) tumor-tissue ECM interactions and associated physicochemical signaling is critical to the development of more pathophysiologically relevant and predictive *in vitro* models^4,5^. For PDAC, in particular, deposition of highly-crosslinked, fibrillar type I collagen by cancer associated fibroblasts (CAFs), also known as desmoplasia, represents a prominent ECM-associated change that has been implicated as a promoter of metastasis and a negative prognostic indicator^6^. Altogether, this points to a need for next-generation preclinical tumor-tissue invasion models that effectively recreate key features of the metastatic phenotype and this desmoplastic microenvironment.

When developing next-generation *in vitro* phenotypic models of tumor invasion, a number of design criteria should be considered. Specifically, while there is advocacy that added model complexity (inclusion of vasculature, various stromal and immune cells) may increase pathophysiologic relevance and predictive power, such approaches fall short with respect to practical logistics^7–9^. For such models to gain traction and widespread use within both pharmaceutical and academic environments, they must be user-friendly, time-efficient, reproducible within and between laboratories, standardizable, scalable, and ideally, amenable to high-throughput (HT) automation (e.g. automated imaging systems, liquid handling robots)^7,10^. Additionally, to achieve their full potential, *in vitro* phenotypic models should move beyond single population-level outcome measures, such as cytotoxicity, and incorporate high-content (HC) multiplex analyses of various relevant cellular processes and behaviors^10,11^. Much work has been done with traditional 3D multicellular spheroid models to demonstrate and improve the relevance of these 3D models over 2D culture^12–14^, to assess the delivery and efficacy of therapeutics^15,16^, and to enable HT-HC screening through standardization and automation^17–19^. However, at present, few, if any, phenotypic invasion models achieve an appropriate balance between pathophysiologic relevance and practical considerations that enable translation to HT-HC screening^7,8^. Finally, since there remains a paucity of model standardization and validation in the published literature, phenotypic model readouts must be correlated with *in vivo* or clinical outcomes to effectively define their predictive power and accuracy^20,21^.

Conventional *in vitro* migration/invasion models (summarized in Table 1) include transwell (Boyden chambers) assays, scratch or exclusion-zone assays, and 3D spheroid invasion assays^22^. Scratch and transwell assays are commonly used because of ease of use, although their geometry, artificial constraints, and general lack of ECM limit their physiologic relevance^22^. 3D spheroid invasion models, where multicellular tumor spheroids are embedded within various 3D matrices, are becoming increasingly common^22–24^. However, adaptation and adoption of these models for HT-HC screens has been hampered by lack of standardization of both spheroid and matrix components and challenges associated with scaling. Other commonly cited shortcomings include: (1) time consuming nature of spheroid creation^10,22^; (2) lack of user control of spheroid size, shape, and cell density^25,26^; (3) reproducibility issues and questionable relevance of 3D matrix component^27,28^; and (4) lack of rapid, reproducible methods for spheroid embedment^26^.

**Table 1:** Comparison of common *in vitro* invasion/migration models.

Criteria for relevant and translatable *in vitro* models		Scratch or exclusion zone	Transwell invasion	3D spheroid invasion	3D tumor-tissue invasion model
Relevance to metastasis	Tumor architecture and geometry	Cell monolayer on 2D tissue-culture plastic	Cell monolayer on 2D transwell insert	Self-aggregated multicellular spheroid embedded in 3D gel/matrix	User-defined tumor compartment embedded in 3D matrix
	ECM microstructure	thin layer or coating	thin layer or coating	3D hydrogel or fibrillar collagen (monomer)	3D fibrillar collagen (Oligomer)
	Accommodates heterogenous cell interactions	no	yes	yes	yes
	Aspect of metastasis being modeled	migration	migration or invasion	invasion	invasion
Reproducible and standardizable setup	Setup time	minutes	minutes	hours to days	minutes
	Reproducible setup	yes	yes	no	yes
	User control of biophysical properties	none	limited tunablity of surroundings	moderate tunablity of surroundings	high tunability of tumor and surroundings
Potential for HT-HC screening	Accommodates automated imaging and analysis	yes	no	no	yes
	Measures multiple phenotypic readouts	no	no	no	yes

**Figure 1:**
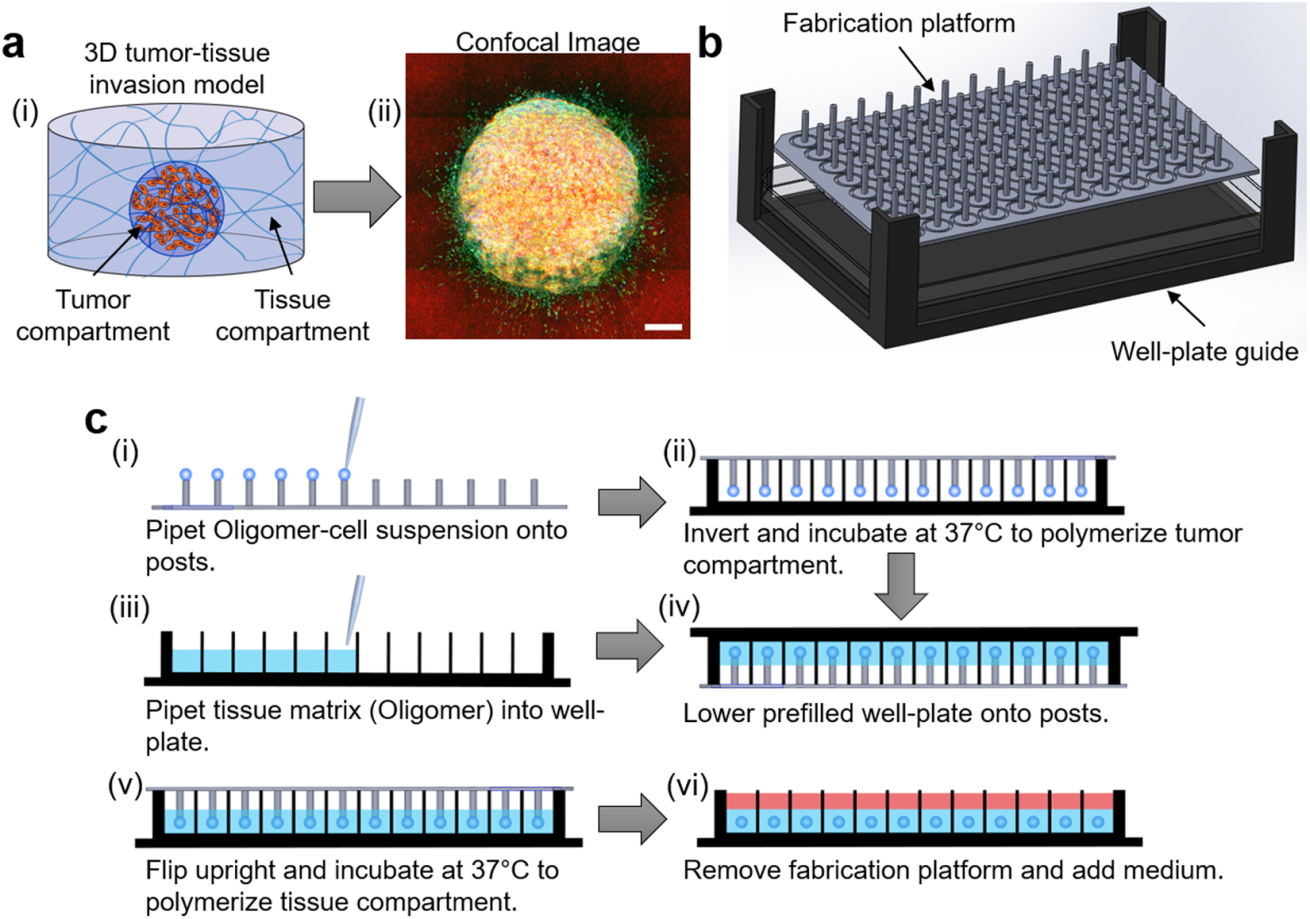
Overview of 3D tumor-tissue invasion model and fabrication system. (**a**) (i) Schematic of 3D tumor-tissue invasion model and (ii) a representative image of tumor cells (Panc-1) invading into the surrounding matrix. Image represents 16 fields of view, each of which is a maximum projection of a 400 μm z-stack (20 μm step; 21 slices) after 5 days of culture; green = actin (phalloidin), blue = nuclei (Hoechst 33342), and red = fibrillar collagen (confocal reflectance). Scale bar = 400 μm (**b**) CAD drawing of custom-designed fabrication system used for rapid and reproducible model setup. (**c**) Process diagram of model setup. (i) Tumor cells suspended in Oligomer are pipetted (5 μL) onto the posts of the fabrication platform using a multi-channel pipet. (ii) Posts are covered with a 96-well plate, inverted, and incubated at 37 °C to induce Oligomer polymerization. (iii) Wells of a second 96-well plate are filled with Oligomer solution. (iv) After tumor compartment polymerization, the prefilled well plate is inverted and lowered onto the posts. (v) The plate is then flipped upright and incubated at 37 °C to polymerize tissue compartment. (vi) Fabrication platform is removed, and culture medium is added. Note: well-plate guide facilitates positioning of the well-plates over the posts of the fabrication platform.

To address the above-mentioned gaps, the goal of this work was to develop a HT-HC phenotypic screening model of PDAC invasion that supports user specification and control of both the tumor and the surrounding tissue compartment, improved pathophysiologic relevance in terms of cell-ECM interactions, and rapid, low-cost implementation. The proposed 3D tumor-tissue invasion model (Fig. 1a) was inspired, in part, by a “multi-tissue interface” model developed for vasculogenesis/angiogenesis studies and involved creation of two adjacent, independently tunable tissue compartments^29^. The use of standardized self-assembling oligomeric type I collagen (Oligomer) for creation of the interstitial ECM supports definition, customization, and standardization of relevant physicochemical parameters, including molecular composition, intermolecular crosslink content, fibril architecture, and matrix stiffness. To adapt and extend this model for HT-HC phenotypic screening, a custom-designed, 96-well fabrication system was created and optimized (Fig. 1b). This low-cost, 3D-printed platform supports precise definition and placement of the tumor compartment within the surrounding tissue compartment (Fig. 1c), which is essential for model interface with automated 3D image collection instruments. This work presents initial development and validation of this new 3D tumor-tissue invasion model which includes demonstrating pathophysiologically relevant modes of invasion by established PDAC cell lines, application of patient-derived PDAC cells and CAFs to recreate more complex heterogeneous cell-cell interactions and invasion mechanisms, and proof-of-concept (POC) drug dosing with image-based multiplex analysis of tumor cell proliferation, metabolic activity, and invasion.

## Results

### Custom fabrication system supports rapid, reproducible tumor-tissue invasion model setup

Since cost, complexity and compatibility with HT screening instruments are viewed as key barriers to more widespread adoption of advanced 3D models^30^, our initial efforts focused on the design of a low-cost fabrication system that facilitated rapid and reproducible tumor-tissue invasion model creation within standard 96-well plates. As shown in Fig. 1, the 3D printed system comprised a well-plate guide and fabrication platform consisting of an array of posts spaced 9 mm apart to match standard 96-well plate specifications (Supplemental Fig. S1). Post tips were concave-shaped to provide the necessary surface area and surface tension for creation, adhesion, and transfer of 5-µL liquid droplets. Post diameter and length, 1.8 mm and 9.9 mm respectively, were optimized so that the tumor compartment could be reproducibly and precisely positioned within the center of each well, 1 mm from the well bottom, in user-friendly fashion. This precise positioning of the tumor compartment (5 µL) within the surrounding tissue compartment (100 µL) facilitates automated collection and analysis of 3D confocal image stacks from the same region within each tumor-tissue construct. When used in conjunction with a multi-channel pipette, this platform supported rapid model setup (a full 96-well plate within 30 minutes) by various users.

### Tumor-tissue invasion model supports customization and standardization of tumor and tissue ECM physicochemical properties

Tumor invasion represents a complex, dynamic process which depends not only upon heterogeneous cell-cell interactions and soluble factor signaling but also ECM make-up, architecture and mechanics^4,31,32^. As such, the ability to define, tune, and standardize relevant ECM composition and biophysical properties in 3D models is essential for mechanistic studies and drug screening, as well as intra- and inter-laboratory reproducibility of model results^33^. Table 2 compares ECM formulations commonly used for invasion models, namely basement membrane extracts (e.g., Matrigel), monomeric type I collagen (atelocollagen and telocollagen), and type I collagen Oligomers. As shown in Table 2, specific advantages of Oligomer include its defined molecular composition and purity, standardized polymerization (self-assembly) specified by starting Oligomer concentration and rheometry-based stiffness of the polymerized matrix, and broad range of tunable matrix stiffness^34,35^. Unlike conventional collagen monomer formulations, Oligomer preserves the telopeptide ends of the collagen molecule, as well as its natural intermolecular crosslinks, which are known to (i) drive collagen fibrillogenesis and self-assembly, (ii) provide resistance against rapid proteolytic degradation, and (iii) most importantly, be prevalent in mature tissues and tumor microenvironments^36–39^.

**Table 2:** Common 3D culture materials used for migration/invasion models.

Characteristic	Basement Membrane Extracts (e.g. Matrigel, Cultrex)	Monomeric Type I Collagen	Oligomeric Type I Collagen
Source	EHS mouse tumor	rat tail or bovine tendon^a^	pig dermis^a^
Primary component(s)	laminin, type IV collagen	type I collagen monomers	type I collagen oligomers
3D structure	non-fibrillar homogenous gel	entanglement of long fibrils with little to no mature intermolecular crosslinks	highly interconnected fibril matrix with mature intermolecular crosslinks
User tunabilty	poor	moderate	good
Mechanical stability	poor	poor	good
Range of achievable stiffness^b^	100%: 100 Pa^c^	1–4 mg/ml: 9–343 Pa^d^	1–4 mg/ml: 27–1440 Pa^d^
Standardization	overall protein concentration	collagen concentration	collagen concentration and shear storage modulus^e^

^a^can derived from other tissues, these are just common examples; ^b^shear storage modulus; ^c^ref.^47^; ^d^ref.^34^; ^e^ASTM International Standard F3089-14 (ref.^88^).

**Figure 2:**
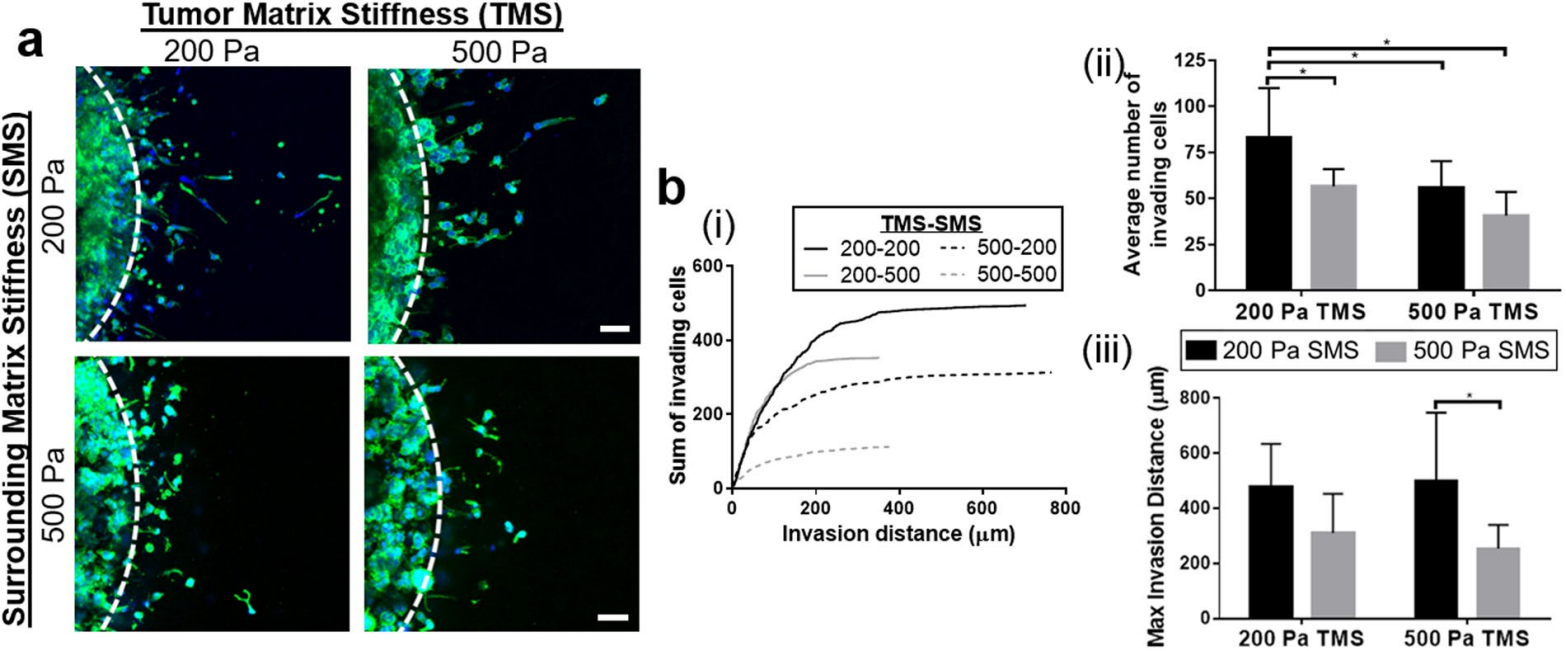
Surrounding matrix density (stiffness) is a primary determinant of tumor cell invasiveness. (**a**) The 3D tumor-tissue invasion model was created using 200 and 500 Pa Oligomer for both the tumor and surrounding tissue compartments. Tumor compartments were prepared with 1 × 10^7^ Panc-1 cells/mL in Oligomer and cultured 5 days. Images represent maximum projections of 150 μm confocal z-stacks; green = actin, blue = nuclei; scale bars = 100 μm. White dotted line represents boundary between tumor and surroundings. (**b**) Quantified tumor invasion is displayed as plots of (i) representative cumulative distribution of all invading cells from a single experiment, (ii) average number of invading cells, and (iii) maximum invasion distance. Bars represent mean ± SD (N = 3, n = 3); asterisks denote statistically different groups (p < 0.05). Legend denoting color-coding of bars applies to (ii) and (iii).

In the present work, collagen fibril density of the tumor and surrounding tissue compartments were independently varied to define how fibril density affected PDAC phenotype and invasion as well as identify model parameters that allowed analysis of PDAC invasion within a 3- to 5-day time period. Oligomer formulations, representing 1.5 and 2.3 mg/ml were used, which corresponded to rheometric-determined stiffness values of 200 and 500 Pa. Panc-1 cells were selected for these studies since they represent an established PDAC line that is known to be invasive, both *in vitro* and *in vivo*^40,41^. As shown in Fig. 2, both the number and maximum distance of invading Panc-1 cells decreased as the stiffness of the surrounding tissue compartment increased. By contrast, tumor matrix stiffness appeared to primarily affect the number of invading cells, with increased stiffness yielding a decrease in invasion cell number. Results showed that use of 200 Pa Oligomer for both the tumor and surrounding tissue compartment yielded the greatest level of invasion over a 5-day period. As such, these model conditions were selected for all subsequent experiments.

### 3D tumor-tissue invasion model recreates expected phenotype-dependent modes of tumor invasion

PDAC invasion *in vivo* is a dynamic and plastic process where cells use a variety of invasion strategies to navigate through the surrounding tissue^27,32,42,43^. While mechanisms underlying these different invasive phenotypes are not fully understood, it is known that tumor cells employ various collective or single-cell invasion modalities based on their epithelial-to-mesenchymal transition (EMT) phenotype and the balance of cell-cell and cell-ECM adhesions^44–46^. To demonstrate that our model can distinguish different invasive phenotypes, two established PDAC cell lines, Panc-1 and BxPC-3 were compared. It is well established, by our laboratory and others, that Panc-1 and BxPC-3 have protein expression and behavior profiles consistent with mesenchymal and epithelial phenotypes, respectively^40,47–49^. Furthermore, while both lines express collagen-binding receptors, including α_2_β_1_ integrin^48^, only BxPC-3 expresses high levels of E-cadherin, a cell-cell junction protein thought to be necessary for collective invasion^50^.

**Figure 3:**
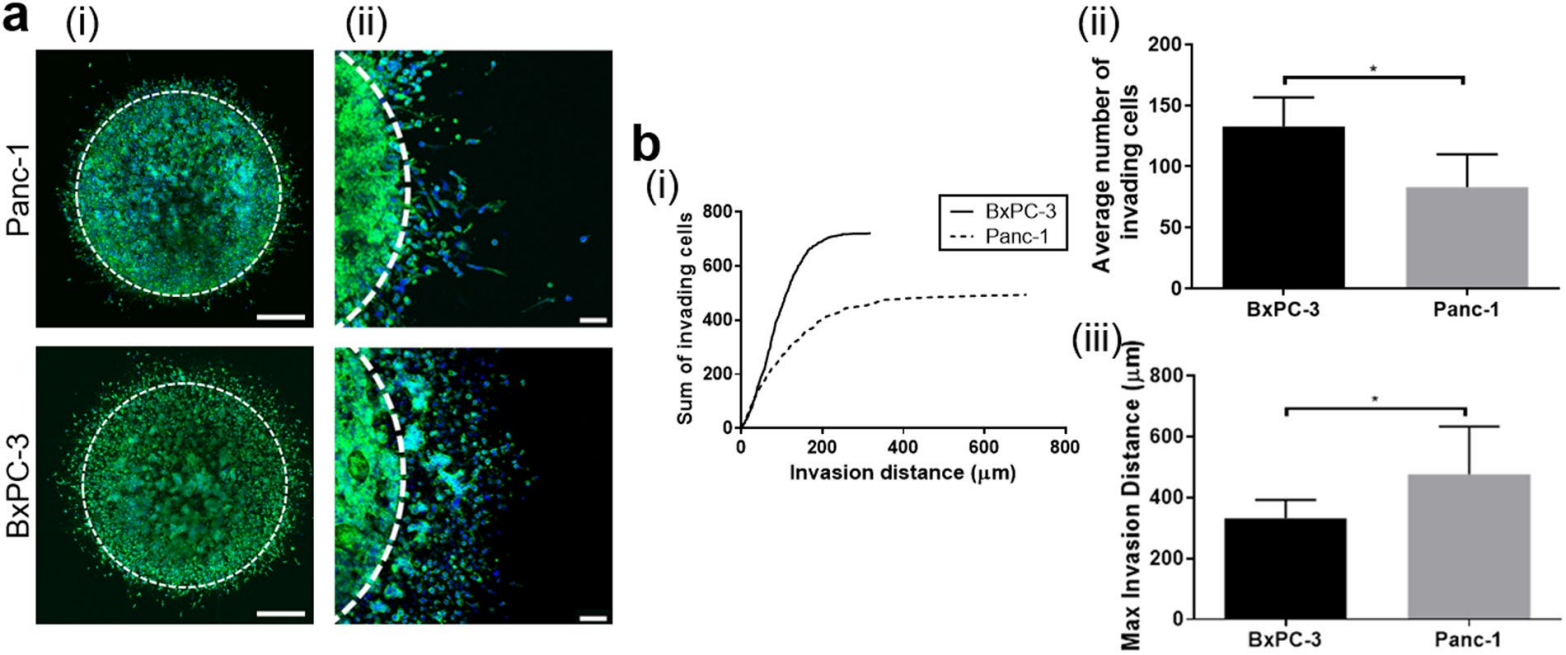
PDAC cells show phenotype-dependent invasion profiles. (**a**) The 3D tumor-tissue invasion model was created using 200 Pa Oligomer for both the tumor and surrounding tissue compartments. Tumor compartments were prepared with 1 × 10^7^ Panc-1 or BxPC-3 cells/mL in Oligomer and cultured 5 days. Images represent either (i) 16 fields of view, each of which is a maximum projection of a 400 μm z-stack (scale bar = 400 μm) or (ii) a single maximum projection of 150 μm z-stack (scale bare = 100 μm); green = actin, blue = nuclei. White dotted line represents boundary between tumor and surrounding compartment. (**b**) Quantified tumor invasion is displayed as plots of (i) representative cumulative distribution of all invading cells from a single experiment, (ii) average number of invading cells, and (iii) maximum invasion distance. Bars represent mean ± SD (N = 3, n = 3); asterisks denote statistically different groups (p < 0.05).

**Figure 4:**
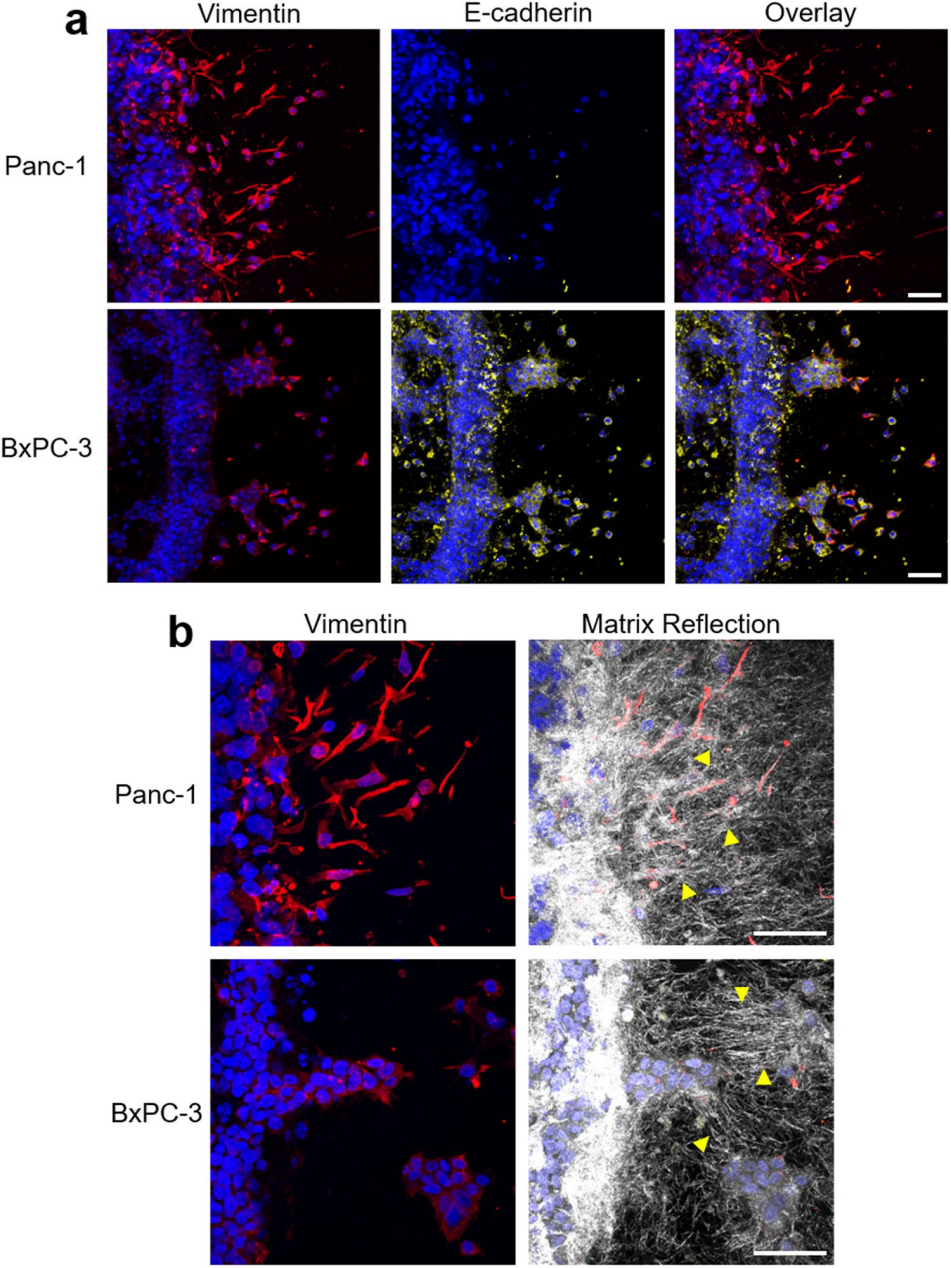
PDAC cells demonstrate different invasive phenotypes. The 3D tumor-tissue invasion model was created using 200 Pa Oligomer for both the tumor and surrounding tissue compartments. Tumor compartments were prepared with 1 × 10^7^ Panc-1 or BxPC-3 cells/mL in Oligomer and cultured 5 days. Constructs were fixed, cryosectioned, and immunostained for (**a**) vimentin (red) and E-cadherin (yellow) with nuclear counterstaining (blue; Hoechst 33342). Images represent maximum projections of 20 μm confocal z-stacks. (**b**) Sections were stained for vimentin (red) and imaged with confocal reflection microscopy to visualize matrix microstructure. Images represent maximum projections of 10 μm confocal z-stacks. Yellow arrowheads denote matrix remodeling and alignment. Scale bars = 50 μm.

As expected, Panc-1 invaded primarily as individual spindle-shaped cells, while BxPC-3 demonstrated collective-cell invasion with a leading front of single-cell invasion (Fig. 3a). Although a greater number (1.6-fold, p < 0.05) of BxPC-3 cells invaded the surrounding tissue, their maximum invasion distance was significantly (1.4-fold, p < 0.05) less than that of Panc-1 (Fig. 3b). Immunostaining further revealed that Panc-1 invasion was mesenchymal in nature with distinct spindle-shaped morphologies, prominent vimentin, and no E-cadherin expression (Fig. 4a). Confocal reflectance microscopy revealed substantial matrix remodeling associated with Panc-1 invasion, as indicated by uniform radial alignment of fibrils perpendicular to the tumor compartment boundary. By contrast, the majority of BxPC-3 cells maintained prominent E-cadherin expression, with a subpopulation of vimentin-expressing cells found at the tumor-tissue boundary and invading the tissue compartment (Fig. 4a). Less matrix reorganization was observed with BxPC-3 with fibril alignment limited to regions between larger aggregates of invading cells (Fig. 4b). Finally, based on western blots, Panc-1 and BxPC-3 embedded within 3D Oligomer appeared to downregulate β-catenin and ZO-1 compared to those grown on 2D tissue culture plastic (Supplemental Fig S2). Western blots also reveal upregulation of vimentin and N-cadherin for Panc-1 in Oligomer, with little discernable changes in E-cadherin for either cell type. Altogether, these results show that our model can distinguish phenotypically-different PDAC tumor cells, as well as EMT-dependent modes of invasion.

### Incorporation of low passage patient-derived PDAC cells and CAFs recreates pathophysiologically relevant heterogeneous cell interactions and EMT-independent invasion

**Figure 5:**
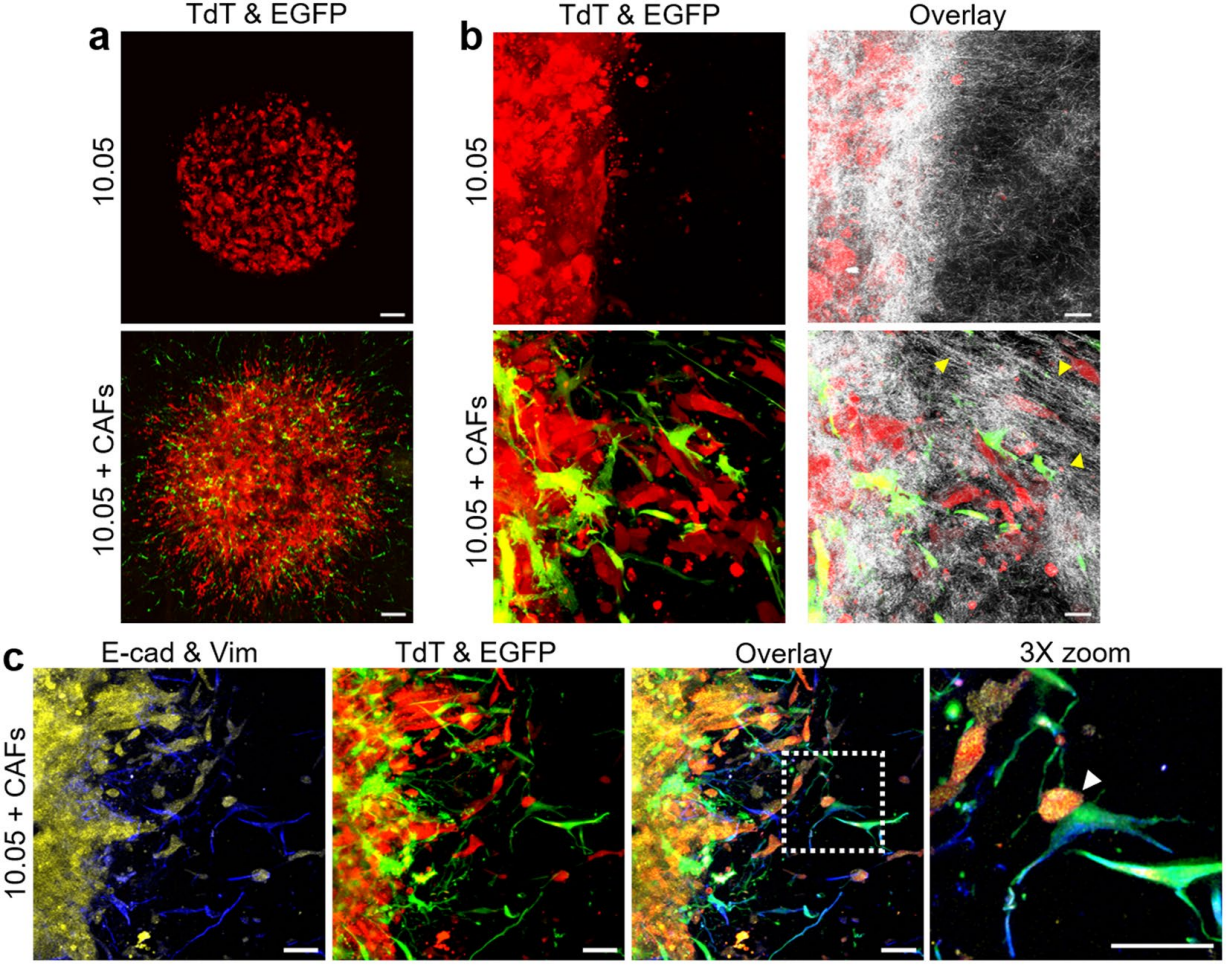
CAFs enhance invasiveness of patient-derived PDAC cells. The 3D tumor-tissue invasion model was prepared with 200 Pa Oligomer for both the tumor and surrounding tissue compartments. Tumor compartments were created with 10.05 alone or 10.05 + CAFS (at 1:1 ratio) at 1 × 10^7^ cells/mL in Oligomer and cultured 4 days. (**a**) Images represent nine fields of view, each of which is a maximum projection of a 400 μm confocal z-stack. Red = tumor cells (TdT) and green = CAFs (EGFP). Scale bars = 200 μm. (**b**) Images represent maximum projections of 10 μm confocal z-stacks from cryosectioned constructs. Confocal reflection microscopy (white) was used to visualize matrix microstructure. Yellow arrowheads denote matrix alignment and remodeling; scale bars = 20 μm. (**c**) Images represent maximum projections of a 20 μm confocal z-stacks of cryosectioned constructs stained for E-cadherin (yellow) and vimentin (blue). Final panel represents a 3X zoom of boxed region in overlay panel. White arrowhead denotes direct interaction between tumor cell and CAF; scale bars = 50 μm.

The cell interactions between PDAC cells and CAFs are known to guide tumor progression, metastasis, and chemoresistance^51–53^, and are thus vitally important for accurate recreation of the tumor microenvironment *in vitro*^8^. To verify that our 3D tumor-tissue invasion model could accommodate this increased complexity and provide additional mechanistic insight into PDAC-CAF interactions, co-culture conditions were established consisting of low-passage, patient-derived PDAC cells (10.05) and CAFs. To aid in visualization and distinction of these two cell populations, 10.05 and CAFs were transfected to express TdTomato Red (TdT) and Enhanced Green Fluorescent Protein (EGFP), respectively (Fig. 5)^54,55^.

When incorporated into tumor compartment without CAFs, 10.05 cells were not invasive, growing as tight clusters (Fig. 5a), and little matrix remodeling was observed except for some matrix densification as indicated by the bright reflection signal immediately adjacent to the tumor compartment (Fig. 5b). Addition of CAFs resulted in a dramatically different phenotype with both 10.05 and CAFs invading the tissue compartment (Fig. 5a) with substantial matrix remodeling, as noted by obvious fibril alignment in the direction of invasion (Fig. 5b). CAFs appeared to physically guide tumor cell invasion, not only by creating a tension gradient in the fibril matrix, but also by altering the cell-cell adhesion balance, as invading 10.05 tumor cells were seen directly interfacing with CAFs (Fig. 5b,c). In terms of EMT protein expression, 10.05 tumor cells maintained prominent E-cadherin, while CAFs stained strongly for vimentin (Fig. 5c). Notably, although some 10.05 cells appeared more elongated, this invasion phenotype maintained E-cadherin expression with no vimentin detected, suggesting either an EMT-independent mode of invasion or the early stages of EMT in which protein expression has not fully switched to the mesenchymal phenotype (Fig. 5c). Overall, these results demonstrate the ability of our model to accommodate heterogeneous cell types relevant to PDAC desmoplasia as well as pathophysiologically relevant EMT-independent invasion.

### 3D tumor-tissue invasion model is amenable to automated HC drug dose screening

Since tumor invasion and metastasis is a complex process involving a variety of biological mechanisms, phenotypic assessment of the effects of therapeutic compounds requires quantification of multiple relevant outcomes. For example, disruption of tumor invasion can occur as a result of decreased tumor cell number, proliferation, motility, or a combination of these^22,56^. However, few, if any, current 3D *in vitro* invasion models have been designed with accompanying multiplex, optical assays to quantify measures of cell health along with relevant invasion metrics for HT-HC screening^22,57^. Therefore, to help fill this gap and more fully understand the effects of chemotherapeutic agents on tumor invasion, we developed a HT-HC assay for quantification of the above-mentioned endpoints.

Additionally, to demonstrate the amenability of our 3D tumor-tissue invasion model for HT-HC phenotypic screens, the multiplex assay was performed using an Opera Phenix High-Content Screening System. Gemcitabine, an analog of deoxycytidine which inhibits DNA synthesis, was selected as the drug of choice since it is clinically relevant in treatment regimens for PDAC patients^58^. The multiplex assay used Hoechst 33342 for nuclear detection and quantification of total cell number and invasion, Click-iT EdU to quantify proliferation, and MitoTracker Red to measure metabolic activity. Panc-1 and BxPC-3 cells were treated for 3 days with a 10-point dilution of gemcitabine, with 20 μM STS as a positive control and 1% DMSO serving as a vehicle control. Representative 3D image stacks of a single 96-well plate row are shown in Fig. 5a, highlighting model reliability and reproducibility for such applications. (See Supplemental Figs S3 and S4 for high magnification images). Note that the dark centers observed in some spheres is an imaging artifact due to imaging depth limitations and does not necessarily indicate a necrotic core.

**Figure 6:**
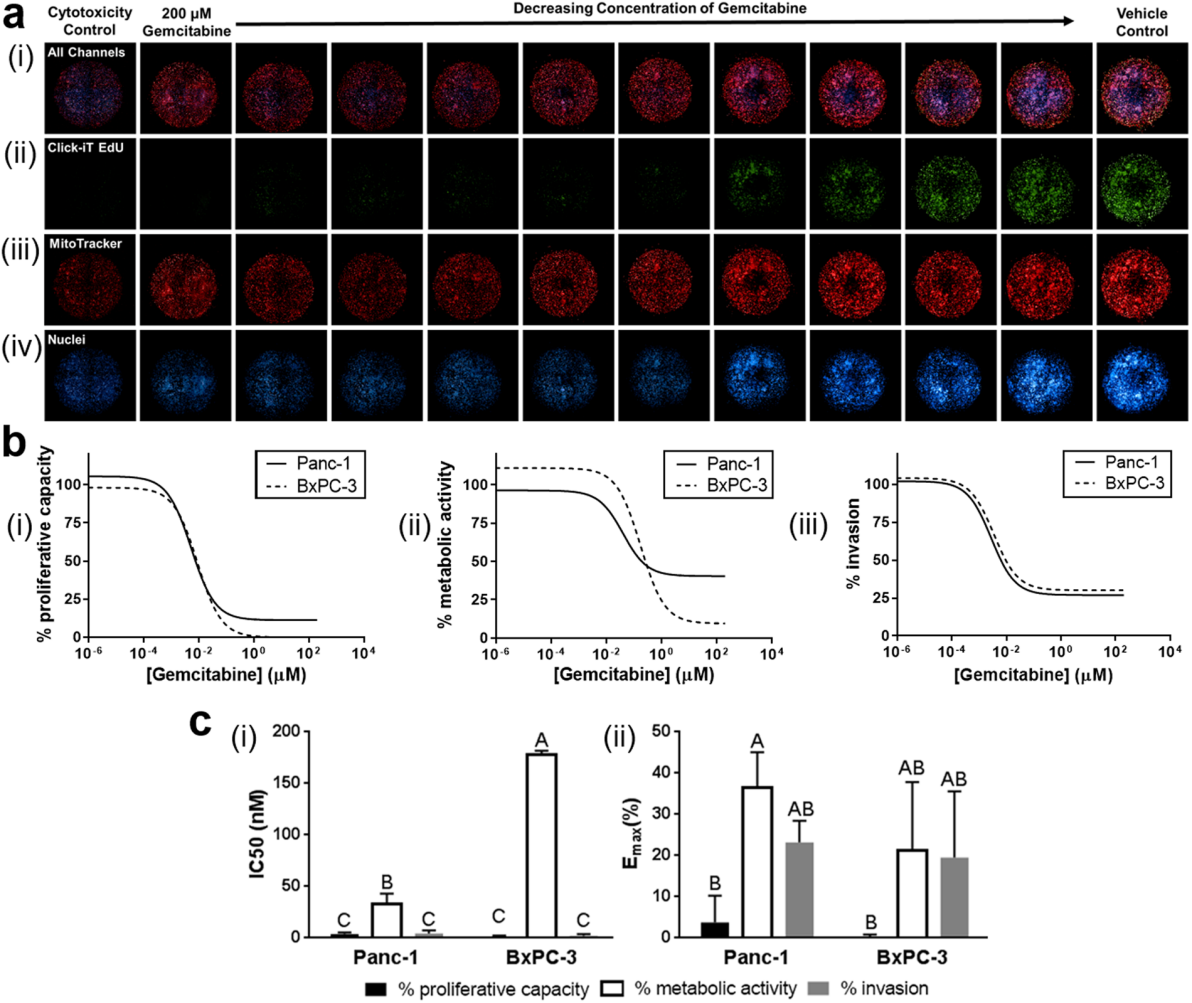
Proof-of-concept drug screen demonstrates HT-HC screening capacity. The 3D tumor-tissue invasion model was prepared with 200 Pa Oligomer for both the tumor and surrounding tissue compartments. Tumor compartments were prepared with 1 × 10^7^ Panc-1 or BxPC-3 cells/mL in Oligomer and treated with serial dilutions of gemcitabine, 20 μM STS (cytotoxicity control), and 1% DMSO (vehicle control) for 4 days. (**a**) Images were obtained using an Opera Phenix. Representative images from a Panc-1 experiment represent maximum projections of 500 μm z-stacks. Columns represent different wells of a 96-well plate; rows represent (i) overlay of all channels, (ii) Click-it EdU 488 stained proliferating cells, (iii) MitoTracker Red stained active mitochondria, and (iv) Hoechst 33342 stained nuclei. (**b**) Representative dose response curves for (i) proliferative capacity, (ii) metabolic activity, and (iii) invasion for Panc-1 and BxPC-3. (**c**) Calculated (i) IC_50_ and (ii) E_max_ values obtained from independent experiments (N = 3) and compared with Tukey-adjusted multiple comparisons. Letters over bars (mean ± SD) denote statistical differences; specifically, bars with the same letter are not statistically different, while those with different letters are statistically different (p < 0.05).

Analysis using Perkin Elmer’s Harmony software facilitated quantification of proliferation, metabolic activity, and invasion as a function of gemcitabine dose (Fig. 6b), as well as associated IC_50_ and E_max_ values (Fig. 6c). Here, relative IC_50_ is used to provide a measure of potency, and E_max_ represents the curve-fit value for the bottom plateau of dose response curves and provides a measure of efficacy. Consistent with gemcitabine’s mechanism of action, IC_50_ and E_max_ values for proliferative capacity were lower than those for metabolic activity and invasion for both Panc-1 and BxPC-3. On the other hand, both IC_50_ and E_max_ for proliferative capacity were not significantly different between cell types, with values ranging from 1–4 nM and 0.3–4%, respectively. While gemcitabine effectively inhibited proliferation, it was found to be less potent and effective at killing tumor cells as indicated by significantly higher IC_50_ and E_max_ values for metabolic activity. Both metabolic IC_50_ and E_max_ values for metabolic activity were greater for BxPC-3 compared to Panc-1; however, only IC_50_ value differences were statistically significant (p < 0.05). Finally, gemcitabine demonstrated only moderate inhibition of invasion in both BxPC-3 and Panc-1 with E_max_ values not going below 25%, indicating incomplete eradication of invasion (Fig. 6b, panel iii). Together these results indicate that gemcitabine is potently and effectively blocking proliferation though not fully killing all cells or stopping invasion of tumor cells, even with concentrations up to 200 μM. Collectively, these results support the utility of this system for HT-HC phenotypic screening with the potential to identify and distinguish new or existing compounds’ effects on proliferation, metabolic activity, and invasion in a single HT-HC assay.

## Discussion

The approval rate of anticancer therapies remains disappointingly low, with recent studies revealing that during the time period from 2004 to 2013, only 7.5% of drugs that entered phase I trials and only 33.2% of drugs that entered phase III trials were eventually approved^59^. These low approval rates, in addition to the exorbitant costs of drug development highlight the need for more efficient and predictive drug development workstreams, that apply predictive phenotypic models to bridge the gap between preclinical and human clinical outcomes^8,57^. In fact, one study using a quantitative decision theory model of pharmaceutical R&D provided evidence showing that the predictive validity of preclinical models should be prioritized over scale-up and cost reduction when working to achieve improved human therapeutic outcomes^60^. Further, even though metastasis and tumor invasion are the main cause of cancer deaths, most approved anti-cancer drugs (approximately 70%) inhibit proliferation or induce cell death, mirroring outcomes measured by traditional drug screening assays^57^. On the other hand, there are few drugs which target invasive phenotypes, in part, because there are few models which appropriately recreate tumor invasion while maintaining the ability to quantify relevant outcomes for high throughput analysis^7,57,61^. *In vitro* 3D phenotypic models and microphysiologic systems are poised to effectively fill this gap in drug development workflows as long as they are developed with the correct design considerations in mind.

Table 1 compares a number of present-day tumor migration/invasion models with regard to some of these important design criteria, revealing a paucity of models which balance pathophysiologic relevance and practical considerations for HT-HC screening^7,8,20,21^. Few of these models have been adapted for use in single-output HT screening, much less multiplex assays for HT-HC screening^57^. For example, Cribbes *et al*. applied multiple spheroid-based assays for evaluation of glioblastoma tumor invasion along with multiple phenotypic outcomes including spheroid size, live/dead staining, and apoptosis (Caspase 3/7). However, these outcomes were all measured in separate assays rather than a single multiplex assay^62^. Others have machined hemispherical pits into well-plate bottoms, facilitating reproducible placement of cell-collagen droplets which were subsequently overlaid with collagen^56^. In this case, monomeric type I collagen was used for creation of tumor droplets and matrix overlay, which required the addition of dialdehyde dextran as an exogenous crosslinker to prevent contraction. Additionally, care was required when differentiating 2D migration from 3D invasion, since tumor droplets were not fully embedded^63^. To date, this example, as well as other 3D invasion models have not been paired with multiplex assays to assess drug sensitivity with multiple phenotypic outcomes^56,64^. In summary, while traditional 2D cultures and floating multicellular spheroids have been paired with multiplex assays for HT-HC phenotypic analyses^18,65,66^, such assay formats have yet to be routinely used in conjunction with invasion/migration models^57^.

Another important differentiating feature between our model and previously published 3D invasion models is the application of oligomeric type I collagen to recreate and tune both the tumor stroma and adjacent tissue compartment. Oligomer molecules retain mature, trivalent intermolecular crosslinks, which are prevalent in mature tissues and have been implicated in tumor invasion and metastasis^37,38,67^. These natural crosslinks modulate the hierarchical packing and self-assembly of collagen-fibril tissues, imparting increased mechanical strength and proteolytic resistance (decreased turnover) without the need for exogenous crosslinking^68^. Thus, Oligomer creates a relevant desmoplasia-like environment and allows for systematic modulation of ECM physical properties such as fibril architecture, stiffness, and proteolytic degradability which can contribute to new mechanistic insight regarding the role of mechanobiology in tumor progression^4^.

While the most relevant mode of human tumor invasion remains unclear^42^, it is noteworthy that the invasive phenotypes observed in this 3D tumor-tissue invasion model align with observations from *in vivo* preclinical models as well as human clinical specimens. The mesenchymal, single-cell invasion exhibited by Panc-1 is consistent with *in vivo* invasion as observed via intravital microscopy within a genetically engineered mouse model of pancreatic cancer with similar genetic mutations (p53 and KRAS)^69^. On the other hand, the collective cell invasion by “tumor buds” and partial EMT exhibited by BxPC-3 in our model, is reminiscent of another relevant invasive phenotype identified in serial sections of human PDAC^42^. Additionally, the cell-line dependent phenotypes observed in this study align with results from both subcutaneous and orthotopic xenograft models in which BxPC-3 tumors remain more epithelial and clustered (differentiated) while Panc-1 tumors are more dispersed (undifferentiated), exhibiting more ECM remodeling, and modestly more invasion^41,70,71^.

When patient-derived cells and CAFs were embedded together within the tumor compartment, it was noted that CAFs greatly enhanced PDAC invasion, as is thought to occur *in vivo*^72,73^. Specifically, heterotypic cell-cell interactions observed in the present work are consistent with observations from human tumor samples and a spheroid invasion model in which heterotypic cadherin junctions form between invading cancer cells (squamous cell carcinoma and lung adenocarcinoma) and CAFs^74^. Additionally, *in vitro* and *in vivo* models of pancreatic and other cancers have shown that CAFs mediate tumor invasion through contractility-dependent matrix remodeling and alignment, similar to the matrix alignment noted in confocal reflection images (Fig. 5b) of the tumor-tissue invasion model^75–77^. Overall, these results with both the PDAC cell lines and the patient-derived PDAC cells and CAFs validate this model’s ability to recreate PDAC invasion and desmoplasia and to accommodate relevant heterogenous cell interactions. To further validate the relevance of this model, future studies are planned with other types of metastatic tumor cells (e.g. breast, lung, bladder) and with cells freshly isolated from patient tumors.

Finally, the multiplex assay and associated results showcase the utility of this 3D tumor-tissue invasion model for predictive HT-HC drug dosing and screening. Observations that gemcitabine is effective at inhibiting proliferation while not fully eradicating the tumor or hindering invasion is consistent with its mechanisms of action as targeting DNA synthesis^58^. Additionally, these results align with those from PDAC xenograft models which show gemcitabine substantially hinders tumor growth and proliferation but does not induce significant apoptosis or reduction of distant metastases and invasion related markers^78–80^. These observations highlight the importance of going beyond traditional screening assays which only assess cell viability or cytotoxicity and move into quantifying multiple phenotypic parameters as we have done here. This type of HC analysis using a 3D phenotypic model opens the door for deeper mechanistic understanding of drugs and more predictive results earlier in the development process^11,81^. While the present work only used one drug and a single set of matrix biophysical properties for model development and proof-of-concept purposes, future studies with this model include validating the HT-HC assay with drugs of different mechanisms of action (e.g. Paclitaxel, Marimastat, Mitomycin C), evaluating novel drug targets (e.g. STAT3, APE-1/Ref-1) and combination therapies, and defining how matrix biophysical properties, namely fibril density and intermolecular crosslinks, modulate drug transport (diffusivity) and cell phenotype, the interplay of which affects the overall drug response. Overall, these strategies, combined with recent advances in computational and animal models have potential to provide a more predictive preclinical portrait of a drug’s efficacy and toxicity to help bridge the gap between preclinical and human outcomes^59,82,83^.

In addition to this model’s potential as a HT-HC drug screening platform, there are many other applications for which it could be used. One such area is the emerging field of “mechanomedicine,” which is focused on identifying and developing therapeutics targeting mechanobiological mechanisms^84^. Because of the high degree of user control of both composition and biophysical properties in both tissue compartments, this tumor-tissue invasion model would be a powerful tool for systematic study of mechanobiology-related mechanisms thought to be involved in tumor invasion and metastasis^4,85^. Specifically, since it is thought that altered matrix stiffness, as mediated through changes in fibril density or intermolecular crosslinking, influences drug sensitivity, future studies will tune these biophysical properties (i.e. fibril density and collagen intermolecular crosslink content) of both tumor and surrounding compartments to characterize how cell-matrix adhesion balance and mechanotransduction guide tumor phenotype, drug transport and drug sensitivity. Transcriptomic data utilizing RNA sequencing could also aid in the mechanistic understanding of the differential expression between cells that remain in the tumor compartment and those that invade outward. Further, because of the size and robust nature of the embedded tumors, this model could be used as an *in vitro* platform to test ablative therapies—something not easily done with traditional 2D culture or spheroids^7^. Finally, with further development and optimization this phenotypic model of invasion could be used as a high-content “culture and sensitivity” screen for personalized medicine^86^.

## Conclusion

With the ever-rising demand for more pathophysiologically relevant and predictive tumor models, it is important to consider how these advanced models will translate and integrate with new or existing workflows. In this work, we aimed to develop a model system which accurately recreated specific features of human cancer while maintaining the ability to quantify relevant outcomes using high-throughput imaging systems. Specifically, our novel 3D tumor-tissue invasion model recreates PDAC desmoplasia and is able to quantify and distinguish various invasion strategies. Additionally, it allows for user customization and standardization; simple, rapid, and reproducible model creation; and automated imaging and analysis to enable HT-HC screening. With continued validation, this 3D tumor-tissue invasion model has great potential to serve as a predictive tool within new preclinical drug development workflows to identify new anti-cancer therapies and decrease the high attrition rates of cancer clinical trials.

## Methods

### Cell culture

Established PDAC cell lines, BxPC-3 and Panc-1, were obtained from American Type Culture Collection (Manassas, VA) and were grown in RPMI-1640 (Life Technologies, Grand Island, NY) and high glucose DMEM (Hyclone, Logan, UT), respectively. Low-passage patient-derived PDAC cells, 10.05, as well as CAFs were grown in high glucose DMEM without sodium pyruvate (Life Technologies)^54,87^. All medium was supplemented with 10% heat-inactivated fetal bovine serum (HI FBS; Life Technologies). Medium for BxPC-3 and Panc-1 was also supplemented with 100 U/mL penicillin and 100 μg/mL streptomycin (Sigma Aldrich, St. Louis, MO), while 10.05 and CAFs were cultured in absence of antibiotics. Cells were maintained in a humidified environment of 5% CO_2_ in air at 37 °C. All cells were passaged at 70–90% confluency; established PDAC lines and CAFs were used below passage 20. Patient-derived PDAC cells were used below passage 10, were authenticated by STR analysis (CellCheck with IDEXX BioResearch) and were tested regularly for mycoplasma contamination.

### Creation of 3D tumor-tissue invasion model

Type I collagen Oligomer was derived from the dermis of market-weight pigs as previously described^34^. Oligomer was dissolved in 0.01 N hydrochloric acid (HCl) and standardized based on molecular composition and polymerization capacity according to ASTM International Standard F3089-14^88^. Here, polymerization capacity refers to the relationship (i.e., polynomial function) between shear storage modulus (G’, Pa) of the self-assembled matrix and Oligomer concentration. To achieve matrices of defined fibril density and matrix stiffness, Oligomer was diluted with 0.01 N HCl to desired concentration and neutralized to physiologic pH with a proprietary 10X Self-Assembly Reagent before the addition of cells. In this study, Oligomer matrices were prepared at stiffness values of 200 and 500 Pa (approximately 1.5 and 2.3 mg/mL, respectively). Validation that the high cell density used for tumor compartment formation did not significantly alter Oligomer self-assembly capacity was performed with 1.5 mg/ml matrices (data not shown).

The model fabrication platform was designed in SolidWorks (Dassault Systemes SolidWorks Corp., Waltham, MA) and 3D printed on a Fortus 400mc 3D Production System (Stratasys, Eden Prairie, MN) using acrylonitrile butadiene styrene (ABS, Statasys). Overall dimensions and post spacing were optimized to accommodate glass-bottom 96-well plates (Cellvis, Mountain View, CA; Supplemental Fig. S1). A well-plate guide was created to aid in uniform and controlled placement of tumor compartments within all wells. The platform and well-plate guide were rendered aseptic by spraying with 80% ethanol and ultraviolet light exposure.

Model setup using the fabrication platform is summarized in Fig. 1c. First, cells were suspended in neutralized Oligomer at 1 × 10^7^ cells/mL, and 5 μL drops were pipetted onto posts with a multi-channel pipette. For co-culture experiments, 10.05 tumor cells and CAFs were combined in the Oligomer-cell suspension at a 10.05 to CAF ratio of 1:1 while maintaining an overall cell concentration of 1 × 10^7^ cells/mL. Once the Oligomer-cell suspension was pipetted onto the posts, the platform was covered with a 96-well plate, inverted and incubated at 37 °C for 8–10 min to allow Oligomer self-assembly. During this incubation time, the wells of another 96-well plate were filled with 100 μL of Oligomer. Once the tumor compartments were polymerized, this prefilled well plate was inverted and lowered onto the platform using the well-plate guide to position the posts in the center of each well and embed tumor compartments within Oligomer. This well plate was then flipped upright and incubated again at 37 °C for 15 min to allow full polymerization of the surrounding matrix. Subsequently, the fabrication platform was removed from the well plate and the appropriate medium added. Experiments comparing Panc-1 and BxPC-3 were cultured for 5 days while drug dosing experiments and those with patient-derived lines were 4 days.

### Analysis of Tumor Cell Phenotype: Invasion and Epithelial to Mesenchymal Transition

For invasion analysis, tissue constructs were fixed with 3% paraformaldehyde (Mallinckrodt, Derbyshire, UK), permeabilized using 0.1% Triton X-100 (Sigma Aldrich), and stained to visualize F-actin (Alex Flour 488 or 546 phalloidin; Life Technologies) and nuclei (Draq5 or Hoechst 33342; Life Technologies). Images were collected using laser scanning confocal microscopy with 10X objectives on either an Olympus IX81 (Olympus, Tokyo, Japan) or a Zeiss LSM 880 (Zeiss, Oberkochen, Germany). To quantify tumor invasion, image analysis was performed on 3D renderings of confocal z-stacks in Imaris (Bitplane, Concord, MA) to obtain number of invading cells and invasion distance (details in Supplemental Methods). Two-factor ANOVA with Tukey-corrected pairwise comparisons (GraphPad Prism, GraphPad Software Inc., San Diego, CA) were used to determine statistical differences (p < 0.05).

To analyze epithelial to mesenchymal transition (EMT), 3D constructs were processed for immunostaining of specific protein markers. After fixation with 3% paraformaldehyde (Mallinckrodt), constructs were soaked in 30% sucrose solution, embedded in Optimum Cutting Temperature (OCT) compound (Fisher Healthcare, Houston, TX), and frozen overnight at −80 °C. Cryosections (60 μm) were prepared using a Thermo Cyrotome FE (Thermo Fisher, Kalamazoo, MI) and Superfrost Plus glass slides (Thermo Scientific). Sections were blocked with 1% bovine serum albumin (BSA; Jackson ImmunoResearch, West Grove, PA) followed by overnight incubation at 4 °C with primary antibodies and 1-hour incubation at room temperature with secondary antibodies. Slides were then rinsed and mounted using Fluro-Gel (Electron Microscopy Sciences, Hatfield, PA). Primary antibodies included mouse anti-vimentin (V6389, Sigma Aldrich) and rabbit anti-E-cadherin (24E10, Cell Signaling Technologies, Danvers, MA). Matched species Alexa Fluor 405, 488, and 633 secondary antibodies (Life Technologies) were used to visualize via immunofluorescence. Slides were counterstained with Hoechst 33342 (Life Technologies) for nuclei identification. Images were collected using laser scanning confocal microscopy with 10X or 20X objectives.

### Proof of Concept (POC) Multiplex Drug Screening Assay

For the POC multiplex drug screening assay, medium containing drugs was added 24 hours after model setup and every 24 hours thereafter for a total treatment time of 72 hours (3 days). Gemcitabine (Alfa Aesar, Tewksbury, MA) was applied as a 10-point drug dilution with a starting concentration of 200 μM and a 1:5 dilution. Staurosporine (Alfa Aesar; 20 μM) and DMSO (Sigma Aldrich; 1%) were used as cytotoxicity and vehicle controls, respectively. For the high-content assay, Click-iT EdU Alexa Fluor 488 (ThermoFisher) and MitoTracker Red CMXRos (ThermoFisher) were used to measure proliferation and metabolic activity. Twenty-four hours prior to fixation, 10 μM 5-ethynyl-2′-deoxyuridine (EdU) and 500 nM MitoTracker were added in serum-free medium along with the final drug treatment. After fixation with 3% paraformaldehyde (Mallinckrodt), constructs were rinsed with 1% BSA (in 1X PBS), permeabilized using 0.1% Triton X-100 (Sigma Aldrich), and incubated overnight at 4 °C with Click-iT reaction cocktail prepared according to manufacturer’s instructions. Finally, constructs were counterstained with Hoescht 33342 (Life Technologies) to visualize nuclei.

Automated confocal imaging was performed using an Opera Phenix High-content Screening System (Perkin Elmer, Waltham, MA). Image analysis was performed in Harmony Software (Perkin Elmer) to evaluate proliferative capacity, metabolic activity, and number of invading cells. (Refer to Supplemental Methods and Supplemental Fig. S5 for further details.) These three measures were each normalized to the cytotoxicity and vehicle controls using the following equation: $\% \,Response=({{\rm{A}}}_{n}-{{\rm{A}}}_{STS})/({{\rm{A}}}_{DMSO}-{A}_{STS})\times 100 \%$. *A*_*n*_ represents the value of the *nth* dilution. *A*_*STS*_ and *A*_*DMSO*_ represent values from the cytotoxicity control and vehicle controls, respectively. These values serve as internal standards to help in normalization of the data from plate-to-plate and within replicates. This data was used to fit three-parameter logistic curves in GraphPad Prism (GraphPad Software Inc.), from which IC_50_ and E_max_ values were calculated. These values were obtained from three independent experiments (N = 3) and compared using a one-factor ANOVA with Tukey-corrected pairwise comparisons to determine statistical differences (p < 0.05).

## Electronic supplementary material

Supplemental Information

## Data Availability

The data generated and analyzed during this study are included in this published article, in its supplementary Information, or are available from the corresponding author on reasonable requests.
