# Supplementary material for: Development of a Novel 3D Tumor-tissue Invasion Model for High-throughput, High-content Phenotypic Drug Screening
Source: Sci Rep. 2018 Aug 29;8:13039. doi: 10.1038/s41598-018-31138-6 (PMC6115445; doi:10.1038/s41598-018-31138-6)
Supplement: Supplementary file 1 — Supplemental Information [file 41598_2018_31138_MOESM1_ESM.pdf]

Development of a Novel 3D Tumor-tissue Invasion Model for High-throughput, High-content Phenotypic Drug Screening

TJ Puls[1], Xiaohong Tan[1], Mahera Husain[1], Catherine F. Whittington[2], Melissa L. Fishel[3,4,5], Sherry L. Voytik-Harbin[1,6,*]

[1]Weldon School of Biomedical Engineering, Purdue University
[2]Department of Oncology, Eli Lilly and Company, Indianapolis, IN 46285
[3]Department of Pediatrics, Wells Center for Pediatric Research, Indiana University School of Medicine
[4]Department of Pharmacology and Toxicology, Indiana University School of Medicine
[5]Pancreatic Cancer Signature Center, Indiana University Simon Cancer Center
[6]Department of Basic Medical Sciences, Purdue University
*corresponding author: harbins@purdue.edu

Supplemental Methods

Details of 3D invasion analysis

For invasion analysis, tumor-tissue constructs were fixed with 3% paraformaldehyde (Mallinckrodt, Derbyshire, UK), permeabilized using 0.1% Triton X-100 (Sigma Aldrich), and stained to visualize actin (Alex Flour 488 or 546 phalloidin; Life Technologies) and nuclei (Draq5 or Hoechst 33342; Life Technologies). Images were collected using laser scanning confocal microscopy with 10x objectives on either an Olympus IX81 inverted microscope with an Olympus Fluoview FV1000 system (Olympus, Tokyo, Japan) or a Zeiss LSM 880 (Zeiss, Oberkochen, Germany). Images were acquired such that the edge of the tumor compartment was along one edge of the image and took up approximately one quarter of the focal volume (850×850×150 μm). To quantify tumor cell invasion, image analysis was performed on 3D renderings of confocal image stacks using the "Cell" analysis package in Imaris (Bitplane, Concord, MA). Briefly, the tumor boundary was defined by thresholding the phalloidin channel to create an object of approximately 2 mm diameter. Nuclei of invading cells were identified by creating 10 μm diameter spots (approximate diameter of average nucleus) using the Draq 5/Hoechst 33342 channel. The distance between the tumor boundary and each nucleus was then calculated and any values less than zero were excluded. Batch processing was used to

analyze all images from a given experiment, followed by manual inspection and adjustment to ensure that intensity thresholds appeared appropriate for each image. Data was used to calculate total number of invading cells, average invasion distance, and maximum invasion distance. Two-factor ANOVA with Tukey-corrected pairwise comparisons (GraphPad Prism, GraphPad Software Inc., San Diego, CA) were used to determine statistical differences ($p < 0.05$).

Details of multiplex assay analysis

After staining for Click-it EdU, Mitotracker Red and Hoescht 33342, automated confocal imaging was performed using an Opera Phenix High-content Screening System (Perkin Elmer, Waltham, MA). Imaging parameters, including exposure time, laser power, and imaging depth were determined based on vehicle control wells and applied to the entire well-plate. To capture the full diameter of the tumor compartment as well as the surrounding matrix, six fields of view from a 10X objective were obtained, with each field representing a 500 µm confocal z-stack (25 µm per step size; 21 slices) which was initiated at the bottom of the tumor compartment. Subsequent image analysis was performed in Harmony Software (Perkin Elmer) using maximum intensity projections of tiled images to evaluate cell proliferation, metabolic activity, and invasion (Supplemental Figure S4). The first step of the analysis process was detection and quantification of all cell nuclei. For invasion analysis, an image region was then created to define the “tumor region.” This was done by blurring the Mitotracker channel with a wide gaussian filter (standard deviation = 10) to smooth out intensity fluctuations within the tumor region while preserving a clear boundary. The “tumor region” was then created by applying an intensity threshold which was adjusted to create a region approximately the same diameter as the whole tumor compartment (Supplemental Figure S4B). The number of invading cells was determined by subtracting the number of nuclei within this “tumor region” from the total number of cells detected in the first step. Proliferative capacity was calculated by detecting and counting all Edu-labeled nuclei and normalizing this number to the total number of nuclei in each image.

To calculate relative metabolic activity, a threshold was applied to the raw Mitotacker image and the resultant fluorescent intensity summed.

Western Blotting

Western blots were used to determine EMT protein expression on a population level for BxPC-3 and Panc-1 in 2D and within 3D Oligomer. Cell lysates from 2D culture were obtained directly from cell culture flasks at 70-80% confluency using chilled 1X RIPA buffer (Millipore, Bedford, Massachusetts) containing 0.2% halt phosphatase and protease inhibitor cocktail (Thermo Fisher Scientific, Waltham, MA), and 2% phenylmethanesulfonyl fluoride solution (Sigma-Aldrich; 2%). Lysates from 3D culture were obtained by snap freezing constructs after four days of culture, grinding them into a powder, and dissolving in lysis buffer. All samples were kept on ice with periodic vortexing for one hour for 2D samples and three hours for 3D samples. Total protein concentration for all samples was determined using a BCA protein analysis kit (Pierce, Rockford, Illinois). Samples containing 30 µg of protein were loaded onto a 4-20% Tris-HCl pre-cast gels (Bio-Rad, Hercules, CA) and transferred onto Trans-Blot Turbo Midi Nitrocellulose membranes (Bio-Rad). After blocking in SEA BLOCK Blocking Buffer (Thermo Fisher Scientific) overnight at 4° C, the membranes were incubated with mouse antibodies against β-catenin (Cell Signaling Technology, 1:1000), E-cadherin (Cell Signaling Technology, 1:1000), ZO-1 (Cell Signaling Technology, 1:1000), N-cadherin (Santa Cruz Biotechnology, 1:1000), and vimentin (BD Biosciences; 1:1000) overnight at 4° C. Mouse antibody against β-actin (Cell Signaling Technology, 1:1000) was used as a loading control. Membranes were then washed in 1X PBS with 0.05% Tween-20 (Sigma-Aldrich) and incubated for two hours at room temperature with horseradish-peroxidase–conjugated IRDye 800CW anti-mouse secondary antibody (LI-COR Biosciences, Lincoln, NE; 1:10000). After multiple washes with the PBS/Tween solution, bands were visualized using Odyssey CLx Infrared Imaging System (LI-COR).

Supplemental Figures

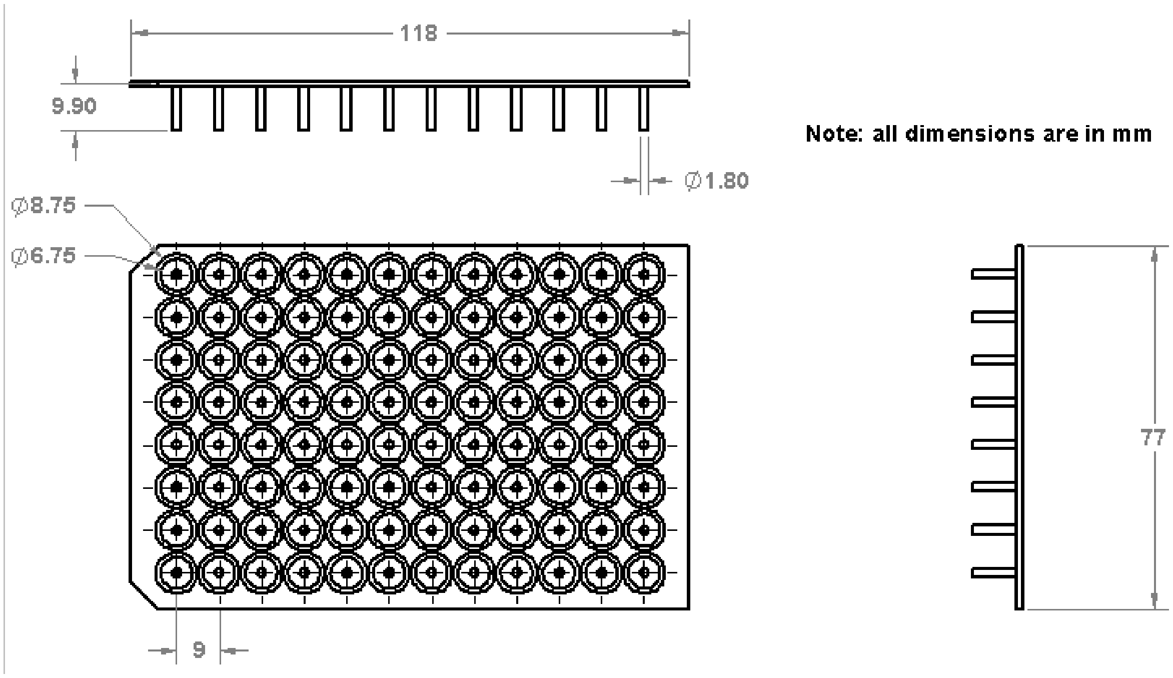

Supplementary Figure S1 – Engineering drawing of fabrication platform

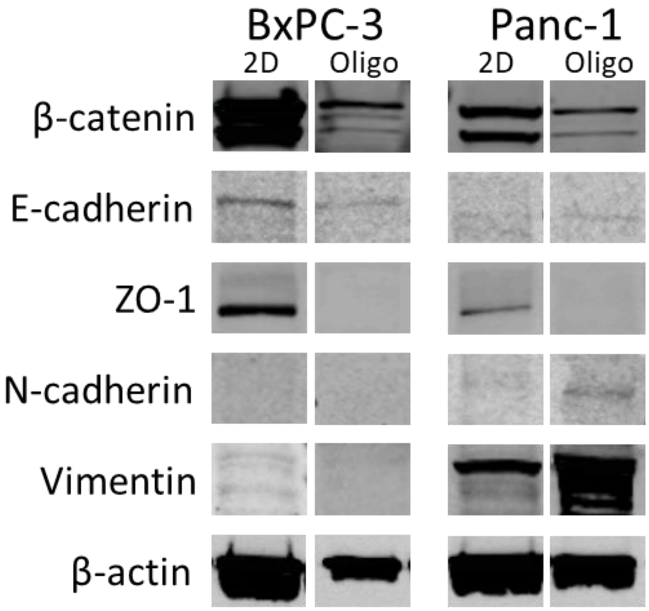

Supplementary Figure S2 – Western blots comparing marker protein expression for PDAC lines when cultured in 2D and 3D Oligomer formats. Western blot protein expression for BxPC-3 and Panc-1 cultured for 4 days on 2D tissue culture plastic or within 3D Oligomer (0.9 mg/ml Oligomer, $2x10^5$ cells/mL). Note: Slightly heavier banding for the housekeeping protein of 2D samples is likely due to residual matrix protein in 3D samples, which effectively decreased the ratio of cellular protein to total protein for 3D samples compared to 2D samples.

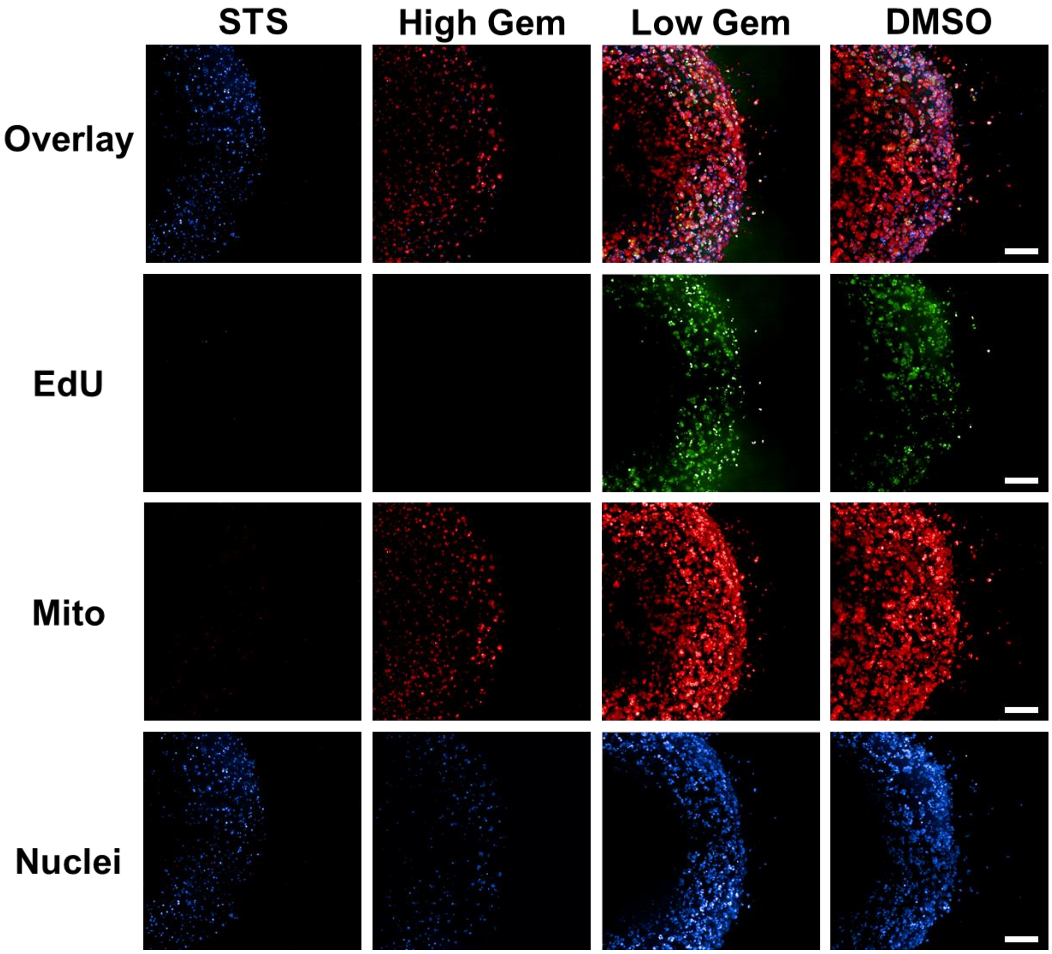

Supplementary Figure S3 – Opera images of controls and high/low gemcitabine doses for Panc-1. The 3D tumor-tissue invasion model was prepared with 200 Pa Oligomer for both the tumor and surrounding tissue compartments. Tumor compartments were prepared with $1x10^7$ Panc-1 cells/mL in Oligomer. Images were obtained using an Opera Phenix and represent maximum projections of 500 µm z-stacks. Columns represent the following different conditions: 20 µM STS (cytotoxicity control), high gemcitabine concentration (200 µM), low gemcitabine concentration (0.10 nM), and 1% DMSO (vehicle control). Rows represent the following: overlay of all channels, Click-it EdU 488 stained proliferating cells, MitoTracker Red stained active mitochondria, and Hoechst 33342 stained nuclei. Scale bars = 200 µm

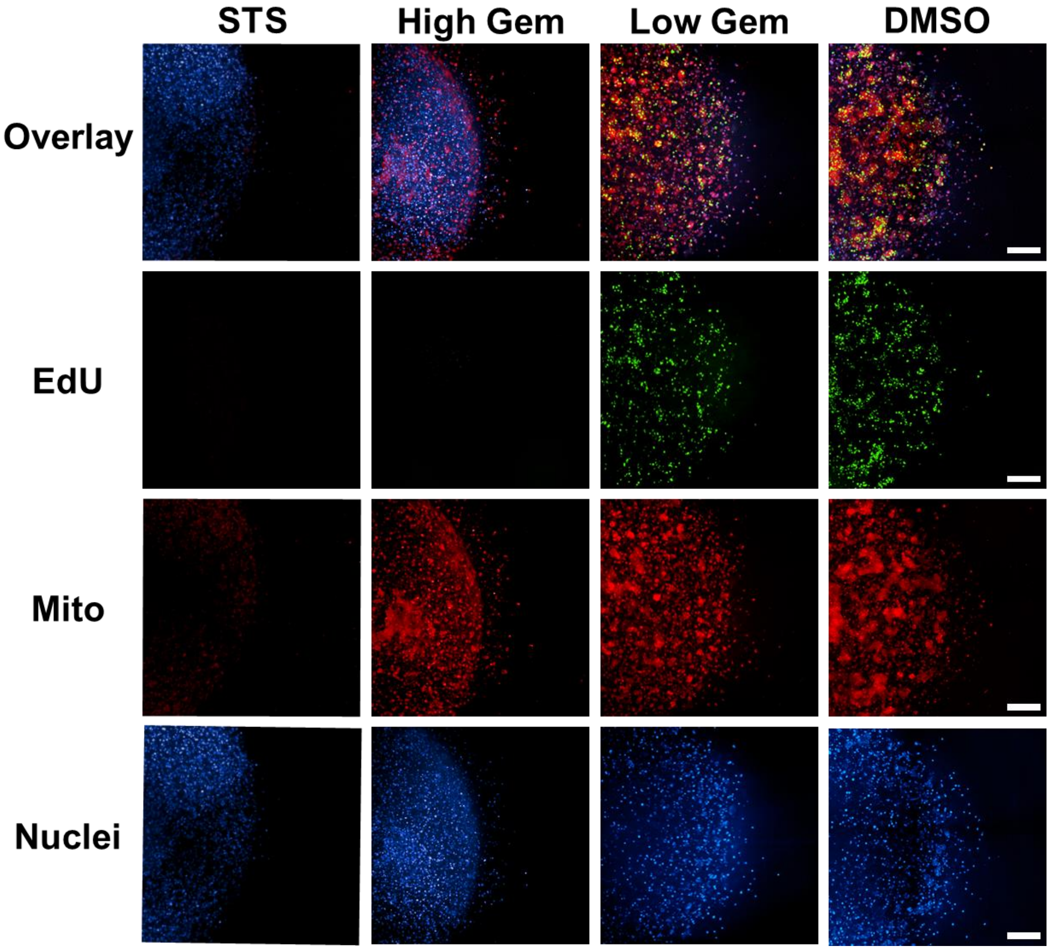

Supplementary Figure S4 – Opera images of controls and high/low gemcitabine doses for BxPC-3. The 3D tumor-tissue invasion model was prepared with 200 Pa Oligomer for both the tumor and surrounding tissue compartment. Tumor compartment were prepared with $1x10^7$ BxPC-3 cells/mL in Oligomer. Images were obtained using an Opera Phenix and represent maximum projections of 500 µm z-stacks. Columns represent the following different conditions: 20 µM STS (cytotoxicity control), high gemcitabine concentration (200 µM), low gemcitabine concentration (0.10 nM), and 1% DMSO (vehicle control). Rows represent the following: overlay of all channels, Click-it EdU 488 stained proliferating cells, MitoTracker Red stained active mitochondria, and Hoechst 33342 stained nuclei. Scale bars = 200 µm

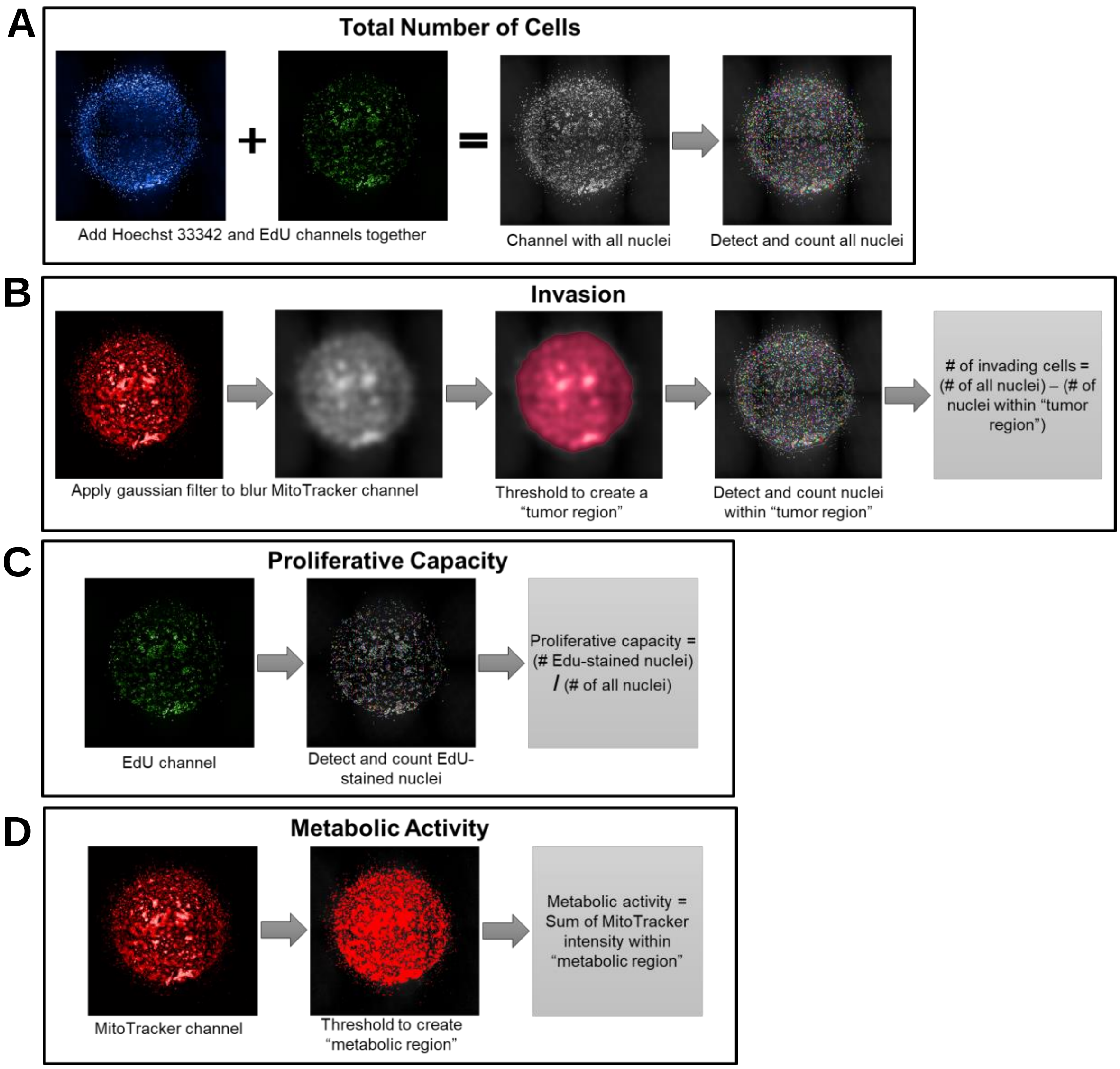

Supplementary Figure S5 – High-content analysis using Harmony software. (A) Determine total cell number by combining Hoechst 33342 and EdU channels and counting all nuclei (B) Calculate the number of invading cells in three steps: 1) create a "tumor region" by blurring and thresholding the MitoTracker channel, 2) count the nuclei within this region, and 3) subtracting the number of cells within the "tumor region" from the total number of cells. (B) Calculate proliferative capacity by counting all EdU-stained nuclei and dividing by the total number of nuclei. (D) Calculate metabolic activity by thresholding the raw MitoTracker channel to obtain a "metabolic region" and summing the intensity of the MitoTracker signal within that region.
